# The first complete mitochondrial genome of *Macalpinomyces bursus* (Ustilaginales: Ustilaginaceae) and insights into its phylogeny

**DOI:** 10.1080/23802359.2021.1944383

**Published:** 2021-08-01

**Authors:** Peng Wang, Tianhao Lu, Jingwei Huang

**Affiliations:** a School of Preclinical Medicine, Chengdu University, Chengdu, Sichuan, P.R. China; b Key Laboratory of Coarse Cereal Processing, Ministry of Agriculture and Rural Affairs, Sichuan Engineering & Technology Research Center of Coarse Cereal Industralization, School of Food and Biological Engineering, Chengdu University, Chengdu, Sichuan, P.R. China

**Keywords:** Mitochondrial genome, phylogenetic analysis, evolution

## Abstract

In the present study, the complete mitochondrial genome of *Macalpinomyces bursus* (Berk.) Vanky 2002 was sequenced and assembled. The complete mitochondrial genome of *M. bursus* was 49,024 bp in length, with the GC content of 30.4%. The *M. bursus* mitochondrial genome contained 27 protein-coding genes, 2 ribosomal RNA (rRNA) genes, and 22 transfer RNA (tRNA) genes. Phylogenetic analysis based on combined mitochondrial gene dataset indicated that the *M. bursus* exhibited a close relationship with species from the genera *Ustilago*, *Sporisorium*, and *Anthracocystis*.

The genus *Macalpinomyces* is a group of plant pathogenic fungi (Li et al. 2017). The three genera, including *Ustilago*, *Sporisorium*, and *Macalpinomyces*, represent an unresolved complex (McTaggart et al. 2012a, 2012b). These smut fungi from *Ustilaginomycotina* contain about 540 described species. Species from the complex often possess characters that occur in more than one genus, creating uncertainty for species placement (McTaggart et al. 2016). Previous studies have shown that it is difficult to identify genus from this complex only by morphology (McTaggart, Shivas, Geering, Callaghan, et al. 2012; Stoll et al. 2005). Mitochondrial genomes have been widely used in the phylogenetic analysis of fungal species (Li, He, et al. 2020; Wang, Song, et al. 2020; Zhang et al. 2017). However, up to now, no complete mitochondrial genome from the genus *Macalpinomyces* has been reported. The complete mitochondrial genome of *Macalpinomyces bursus* (Berk.) Vanky 2002 will promote the understanding of phylogeny, evolution, and taxonomy of this important fungal genus.

The specimen (*M. bursus*) was collected from Yunnan, China (101.24 E; 24.89 N). The specimen was then deposited at Collection Center of Chengdu University (contact information: Jingwei Huang, email: huangjingwei1003@qq.com) under the voucher number of Mbur_s99. The complete mitochondrial genome of *M. bursus* was sequenced and *de novo* assembled according to previous described methods (Li, Ren, et al. 2019; Li, Xiang, et al. 2019; Wang, Song, et al. 2020; Wang, Wang, et al. 2020). First, we extracted the total genomic DNA of *M. bursus* using a Fungal DNA Kit (D3390-00, Omega Bio-Tek, Norcross, GA, USA). Then, the extracted genomic DNA was purified using a Gel Extraction Kit (Omega Bio-Tek, Norcross, GA, USA). The purified DNA was stored in Chengdu University (No. DNA_ Mbur_s99). We constructed sequencing libraries using a NEBNext® Ultra™ II DNA Library Prep Kit (NEB, Beijing, China). Whole genomic sequencing of *M. bursus* was conducted using the Illumina HiSeq 2500 Platform (Illumina, SanDiego, CA). We *de novo* assembled the mitochondrial genome of *M. bursus* using NOVOPlasty v4.3.1 (Dierckxsens et al. 2017; Li, Ren, et al. 2020). The complete mitochondrial genome of *M. bursus* was annotated according to previous described methods (Li et al. 2018; Li, Li, et al. 2021). Briefly, the protein-coding genes, rRNA genes, tRNA genes, and introns of the *M. bursus* mitochondrial genome were annotated using MITOS (Bernt et al. 2013) and MFannot (Valach et al. 2014), both based on the genetic code 4. We also predicted protein-coding genes (PCGs) or open reading frames (ORFs) based on the NCBI ORF Finder (Coordinators 2017), and annotated by BLASTP searches against the NCBI non-redundant protein sequence database (Bleasby and Wootton 1990). The tRNA genes in the *M. bursus* mitogenome were also predicted with tRNAscan-SE v1.3.1 (Lowe and Chan 2016).

The complete mitochondrial genome of *M. bursus* is 49,024 bp in length. The base composition of the *M. bursus* mitochondrial genome is as follows: A (34.15%), T (35.45%), G (15.09%), and C (15.31%). The complete mitochondrial genome of *M. bursus* contains 27 protein-coding genes, 2 ribosomal RNA genes (*rns* and *rnl*), and 22 transfer RNA genes. The genome size and GC content of *M. bursus* were the lowest in the *Ustilago*, *Sporisorium*, and *Macalpinomyces* complex. A total of 6 introns were detected in the PCGs of *M. bursus* mitogenome, including Mbu.cox1P386, Mbu.cox1P709, Mbu.cox1P971, Mbu.cobP393, Mbu.cobP429, and Mbu.cobP490, which were named according to previous studies (Zhang and Zhang 2019). We constructed a phylogenetic tree for 20 basidiomycete species to investigate the phylogenetic status of *M. bursus*. *Turbinellus floccosus* from the order *Gomphales* was set as outgroup (Cheng et al. 2021). The Bayesian analysis (BI) method was used to construct phylogenetic tree based on the combined 14 core protein-coding genes according to previous described methods (Li, Wu, et al. 2021; Li, Yang, et al. 2020). Briefly, single mitochondrial genes were first aligned using MAFFT v7.037 (Katoh et al. 2019), and then, we concatenated these alignments to a gene set using the SequenceMatrix v1.7.8 (Vaidya et al. 2011). Best-fit models of evolution and partitioning schemes for the gene set were determined according to PartitionFinder 2.1.1 (Lanfear et al. 2017). MrBayes v3.2.6 (Ronquist et al. 2012) was used to analyze the phylogenetic relationships of the 20 basidiomycete species based on the combined gene set. As shown in the phylogenetic tree (Figure 1), the mitochondrial genome of *M. bursus* exhibited a close relationship with species from the genera *Ustilago*, *Sporisorium*, and *Anthracocystis*.

**Figure 1.**
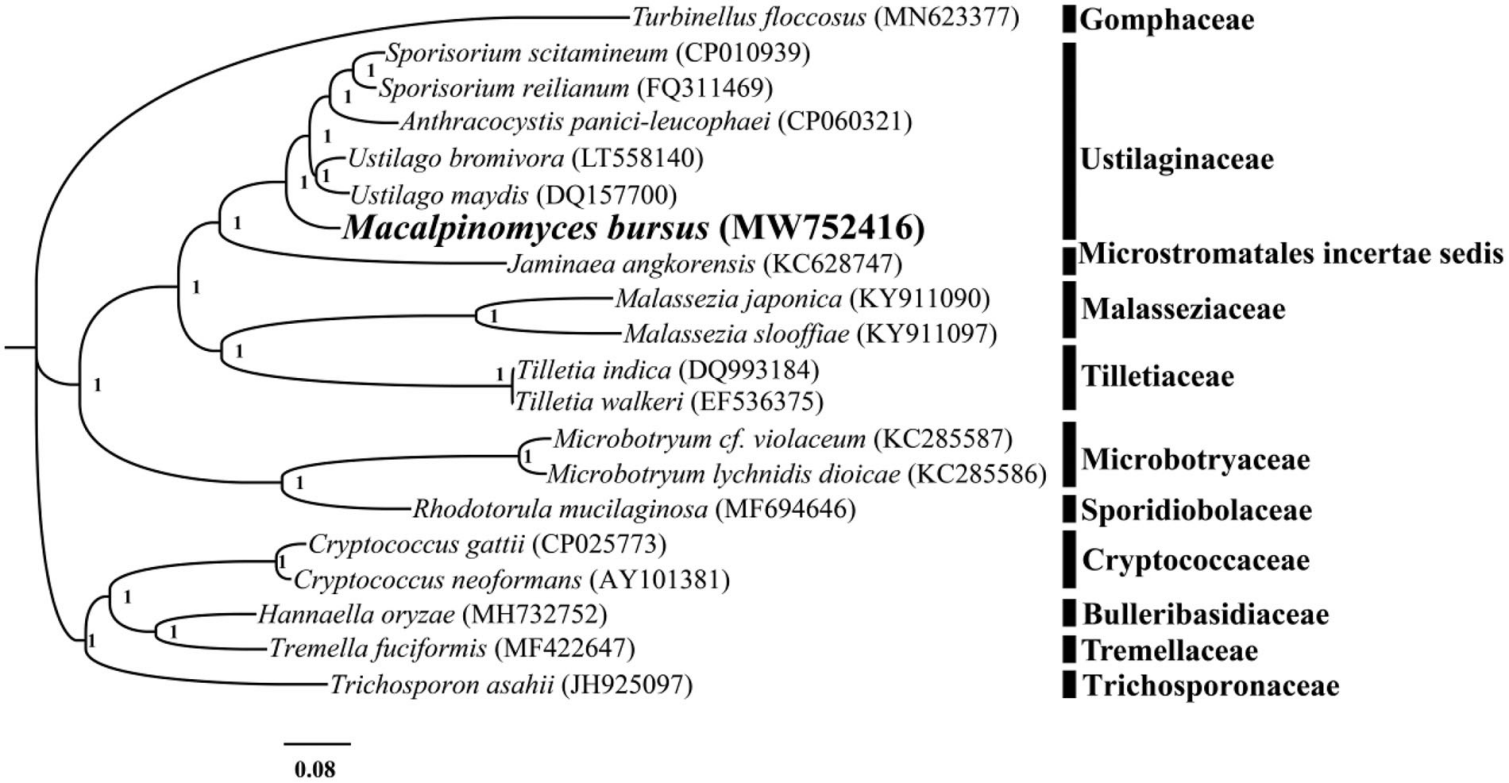
Bayesian phylogenetic analysis of 20 species based on the combined 14 core protein-coding genes. Accession numbers of mitochondrial sequences used in the phylogenetic analysis are listed in brackets after species.

## Data Availability

The genome sequence data that support the findings of this study are openly available in GenBank of NCBI at (https://www.ncbi.nlm.nih.gov/) under the accession no. MW752416. The associated BioProject, SRA, and Bio-Sample numbers are PRJNA724891, SRR14320034, and SAMN18864185, respectively.
